# PharmaTrek: A Semantic Web Explorer for Open Innovation in Multitarget Drug Discovery

**DOI:** 10.1002/minf.201200070

**Published:** 2012-08-07

**Authors:** Maria C Carrascosa, Oriol L Massaguer, Jordi Mestres

**Affiliations:** a Research Programme on Biomedical Informatics (GRIB), IMIM Hospital del Mar Research Institute and University Pompeu Fabra, Parc de Recerca Biomèdica, Doctor Aiguader 88, 08003 Barcelona, Catalonia, Spain

**Keywords:** Semantic web, Chemogenomics, Target profile, Polypharmacology

The realisation that vast amounts of pharmacological data for small molecules are continuously being reported in bibliographic sources has promoted in recent years the rise of initiatives aiming at collecting, organising, and storing these data together with chemical structures. Today, there are numerous databases that connect hundreds of thousands of small molecules to thousands of biological responses of their interaction with macromolecules. Some of these repositories, such as GLIDA, PDSP, BindingDB, IUPHARdb, PubChem, ChEMBL, and DrugBank, make all data available in the public domain.^1^ In addition, some others, such as BioPrint, Integrity, Wombat, and GOSTAR, offer access to their data only through licensing from the respective commercial providers.^2^

This wide diversity of sources does not facilitate direct access and interrogation of the entire contents covered by all of them. Integrating all these repositories into a single accessible resource is not trivial, mainly due to issues related with the use of different vocabularies and ontologies for the various domain entities which makes cross-referencing among multiple sources a challenging task.^3^ But even if some degree of integration is accomplished, managing and updating such an integrated framework in an efficient manner may require significant human resources and be extremely time consuming and difficult to fully automate.^4^ Managing chemical structures, for instance, involves taking into consideration a fair amount of detailed aspects such as salt formulation and isomerism (tautomerism, regioisomerism, and optical and geometrical isomerisms), and it has been reported that different molecular identifiers may actually lead to an essentially different number of unique chemical structures depending on the user criteria for defining uniqueness.^5^ On the other hand, managing pharmacological data across databases is also complicated, as one may encounter different values for the same molecule – protein interaction obtained from different laboratories, from the same protein but different species, or from the same protein and species but different settings and conditions.^6^

In parallel, there have been some recent initiatives to provide some conceptual meaning to the connections established between objects from different domains, so the links are stored in such a way that become more understandable to computers. This is the main goal of applying semantic web technology to drug discovery.^7^ Semantic web,^8^ also known as Web 3.0, is a web of data that provides tools to unify them in a consistent way and gives access to them through standardized query methods. The main difference with the so-called Web 2.0 is that instead of dealing with a huge amount of dispersed data that requires some level of human interpretation to understand it, data is integrated and conceptualised in a way that computers themselves can “understand” and extract new knowledge from them.

Several recent projects have implemented semantic web technologies in a life science environment. Among them, of mention are Bio2RDF,^9^ that codifies the contents of different public biological databases into a resource description framework (RDF), Linking Open Drug Data (LODD),^10^ that makes a similar task but focussed mainly on drug data, and Chem2Bio2RDF,^11^ that integrates small molecule and drug information with protein targets, genes, and pathways, and allows cross-source linking with LODD and Bio2RDF.

Along these lines, Open PHACTS is a recently funded European project that applies semantic web standards and technologies to create an integrated open pharmacological space (OPS) aiming at facilitating open innovation in drug discovery research.^12^ With this semantic approach, Open PHACTS aspires to solve some of the main bottlenecks of current data access and knowledge generation in drug discovery, namely, access to multiple disparate heterogenic information sources, lack of standards and common identifiers for domain entities, and ability to interrogate the system with complex research questions. At present, OPS offers access to ChEMBL v1.3,^13^ one of the largest public repositories of chemical structures annotated with pharmacological data that has recently integrated in it the contents of other individual sources. With respect to identifiers, vocabularies, and ontologies, OPS uses ConceptWiki,^14^ a collaborative knowledge resource for the life sciences that provides a mapping between scientific textual representations of concepts and database and ontology identifiers. Finally, it is envisaged that OPS provides the framework on which external applications may be developed to allow users to address complex research questions to the system and display the results in an interactive environment that facilitates knowledge extraction.

With this purpose in mind, we introduce PharmaTrek (http://cgl.imim.es/pharmatrek), an interactive semantic web explorer purposely designed for researchers in the field of multitarget pharmacology to address complex queries in a most simple and intuitive manner. Access to the RDF {chemical object}—{predicate}—{protein object} triple store of ChEMBL v1.3 is currently managed by an application webserver that retrieves data from OPS through a SPARQL endpoint, but also through an application programming interface (API) provided by the Open PHACTS system. A scheme of the application architecture used is provided in Figure 1.

**1:**
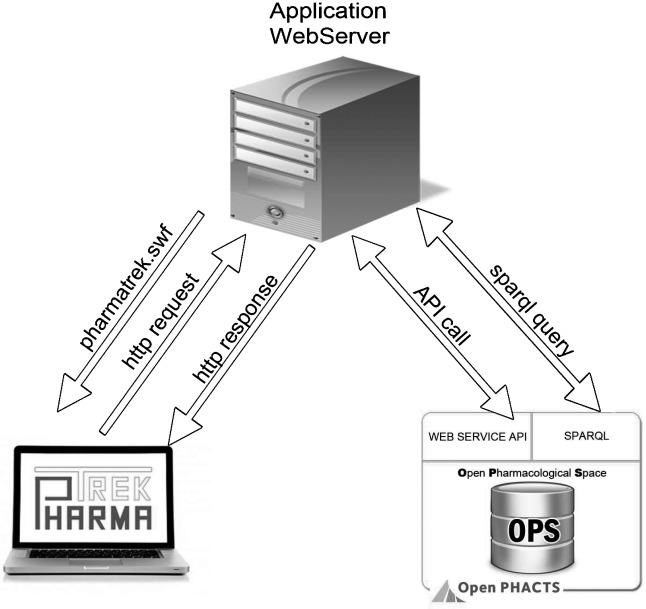
Application architecture.

As an example of the type of complex queries that can be addressed, we will ask PharmaTrek to retrieve all ligands having a -log(Activity) value (Activity being generally defined here as any of the interaction types available in OPS, such as *K*_i_, *K*_d_, *IC*_50_, or *EC*_50_) larger than or equal to 7.5 (that is, more potent than 31.62 nM) for coagulation factor Xa (EC 3.4.21.6) and being at least two orders of magnitude selective against trypsin (EC 3.4.21.4) and thrombin (EC 3.4.21.5), two phylogenetically related serine proteases. Afterwards, we will show how this first query can be further refined by adding other potentially relevant proteins that were not taken into consideration when defining the original target profile.

A typical query in a multitarget drug discovery project requires first to define an objective target profile. In order to do that, type the name of the first target in the prompt of the field located in the upper-left workspace, labeled as “Enter target name”. After typing the first characters of the target name, the prompt will start suggesting names of proteins matching that string. You can then press “Enter” to retrieve the list of suggested names in the space located right below the prompt. To obtain protein name suggestions, the field currently generates a SPARQL query that searches the text entered into the OPS repository. If your intended target appears in the list, you can then simply put the mouse pointer on top the target name and drag and drop it into the target profile basket located on the right hand side. You can then repeat the process for every target in your objective profile. In our case study, the target profile basket should contain the names of Thrombin, Trypsin I, and Coagulation factor X.

Once the target profile has been defined, you can click on the “Show heatmap” button and an interaction map will appear in the largest workspace available. At this stage, it contains 7488 molecules with activity data for any of the three targets in the profile. Now, you can apply affinity filters to each individual target to meet certain selectivity criteria. You can do that by just clicking on the arrow next to the target name. By doing that, two fields will appear that will allow you to enter a minimum and a maximum activity value. In our case, since we are looking for potent and selective factor Xa inhibitors, we will enter a minimum affinity value of 7.5 for Coagulation factor X and maximum affinity values of 5.5 for both Thrombin and Trypsin and we will press the “Show heatmap” button again. Additional filters that affect all protein entries in the target profile basket can be defined in the “General filters” space. There are currently four general filters that can be defined to further refine your queries on protein species, interaction type, and general minim and maximum affinity values.

The results of the query with individual target affinity filters are shown in Figure 2. As can be observed, the number of molecules meeting those affinity criteria has now been reduced to 3966 molecules, represented as rows in the interaction map. Note that summary information on the size of the heatmap (molecules and targets) and overall minimum and maximum affinity values can be found on the right-hand side of the heatmap, above which there is also an interactive small-size overall viewer that allows you to zoom in and out on different regions of the interaction map. The default colour gradation used in the heatmap is green for lack of information about the molecule — protein interaction, yellow for the minimum interaction value, and dark red for the maximum interaction value. Colour gradation is adapted as subsequent filters are applied and new minimum and maximum values are present. For the sake of convenience, one can also customise the colours of the heatmap with the colour selectors that are located at the bottom-right corner of the heatmap.

However, what is currently shown in Figure 2 is still not the final answer to our intended query. To keep only those ligands that have interaction values with all the proteins defined in the target profile, you ought to click on the “connect” check box located right next to the “Show heatmap” button. Effectively, clicking on “connect” applies a logic AND to all targets and filters defined in the basket. This action results in a final number of 101 potent and selective factor *Xa* inhibitors, relative to thrombin and trypsin. Visual quantitative confirmation of potency and selectivity for each molecule, can be obtained by passing the mouse over the interactive heatmap. A tooltip will then appear with the pActivity value of the interaction between a ligand (in the row) and a target (in the column).

**2:**
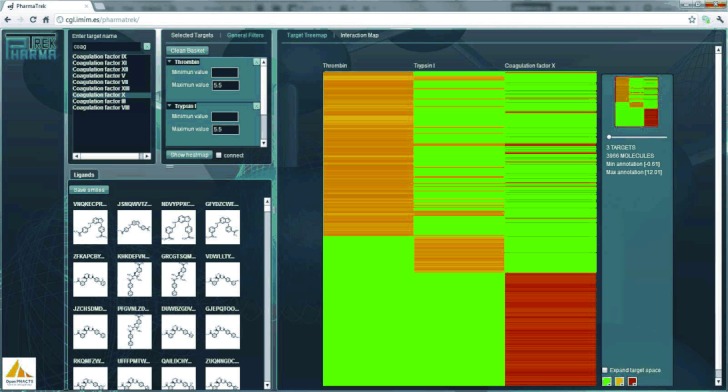
PharmaTrek layout showing the results of a query on ligands having a defined affinity profile on three serine proteases (see text for details).

At this stage, the original question has been answered, and molecules meeting all potency and selectivity criteria across the target profile defined have been identified. However, one could go one step further and check whether any additional targets should be added to the original definition of the target profile on the basis of the information contained in OPS. In the bottom-right corner of the application, you will find an “Expand target space” check box that meets this precise need. By clicking on the “Expand target space” check box, PharmaTrek makes a request to expand the target space of the 101 potent and selective factor *Xa* ligands with any activity data on additional targets not included originally in the target profile defined in the basket. In this particular case, interaction data for 16 additional targets are retrieved. Among them, tissue-type plasminogen activator is one of the targets showing a high degree of cross-pharmacology with the three targets in the original profile. Based on these findings, one may now decide that this target should be included in the objective target profile of the multitarget drug discovery project. To do that, simply put the mouse on top of the target name appearing in the labels of the columns (zoom in if necessary) and drag and drop the name into the target profile basket. You can then apply new filters and perform a new ligand extraction request.

At any point during a PharmaTrek session, the structures of all ligands contained in the interaction map appear in the “Ligands” workspace, right below the target profile basket. By clicking on the image of any chemical structure, a ligand card is displayed with all the information about the ligand present in OPS. One can always save the Smiles of all chemical structures present in the workspace by pressing the “Save smiles” button. This is a useful feature to import any ligand selection to another external application.

We have introduced PharmaTrek v1.0, a semantic web explorer of pharmacological space for open innovation in multitarget drug discovery. Other existing applications, such as SuperTarget, STITCH, DrugViz, and iPHACE, provide means to access and visualise drug-target interactions.^15^ PharmaTrek differs conceptually from those tools by the way the user submits complex multitarget queries to the single largest open pharmacology space available to date (ChEMBL v1.3) and visualises the results in an unique interactive manner that allows taking informed decisions on the original objective multitarget queries. Further development is currently underway in our laboratory.

## Computational Methods

PharmaTrek is implemented using Flex 4.^16^ Flex is a free, open source framework for building and maintaining highly interactive, expressive Rich Internet Applications (RIA) that deploy consistently on all major browsers. Flex uses two languages to write applications: MXML^17^ and ActionScript.^18^ MXML is an XML markup language used mainly to lay out user interface components, but also to implement the visual aspects of an application. ActionScript is an object-oriented programming language. ActionScript 3.0 is designed to facilitate the creation of highly complex applications with large data sets and object-oriented reusable code bases. We also use FlashDevelop^19^ as Integrated Development Environment (IDE) to build our application. FlashDevelop is a free and open source (MIT license) code editor. Finally, PharmaTrek was developed following the Model-View-Controller^20^ design pattern to facilitate the reusability and maintainability of the application. Accordingly, the application is partitioned into three categories of components: model components that encapsulate data and behaviors related to the data processed by the application, view components that define the application’s user interface, and the user’s view of the data, and controller components that handle data interconnectivity in the application.
